# Iodine activates NLRP3 inflammasomes in PBMCs of patients with autoimmune thyroiditis and regulates Th1 and Th17 cell differentiation

**DOI:** 10.1530/EC-24-0456

**Published:** 2025-01-16

**Authors:** Ying Wu, Qingling Guo, Yongping Liu, Xun Gong, Wei Sun, Yushu Li, Chenling Fan, Weiping Teng, Zhongyan Shan

**Affiliations:** ^1^ Department of Endocrinology and Metabolism, Institute of Endocrine, NHC Key Laboratory of Diagnosis and Treatment of Thyroid Diseases, The First Hospital of China Medical University, Shenyang, Liaoning, PR China; ^2^ Department of Endocrinology, The Affiliated Sir Run Run Shaw Hospital, College of Medicine, Zhejiang University, Hangzhou, Zhejiang, PR China; ^3^ Department of Endocrinology, Shandong Provincial Hospital Affiliated to Shandong First Medical University, Jinan, Shandong, PR China; ^4^ Department of Thyroid Surgery, The First Hospital of China Medical University, Shenyang, Liaoning, PR China

**Keywords:** autoimmune thyroiditis, inflammasome, peripheral blood mononuclear cells, iodine, T cell subsets

## Abstract

**Objectives:**

Inflammasomes are associated with various autoimmune diseases. Herein, we aimed to study the occurrence of inflammasomes in peripheral blood mononuclear cells (PBMCs) from patients with autoimmune thyroiditis (AIT), and the relationship between their abundance and the inflammatory response index of AIT. Furthermore, we examined the effect of iodine on inflammasomes containing NLR family pyrin domain-containing 3 (NLRP3) and inflammasome activation of helper T (Th) cell differentiation regulation in cultured PBMCs.

**Methods:**

We collected PBMCs and serum samples from 50 patients with AIT with normal thyroid function and 50 controls matched for age and sex. In PBMCs, the mRNA and protein expressions of certain inflammasome constituents (e.g., NLRP1, NLRP3, absent in melanoma 2 (AIM2) and caspase-1), interleukin (IL)-1β and IL-18 were assessed using qRT-PCR and western blotting. Enzyme-linked immunosorbent assays (ELISAs) assessed the serum levels of IL-1β and IL-18. Flow cytometry was employed to examine NLRP3 expression on CD14^+^ monocytes and Th1 and Th17 cell percentages in the groups. AIT- or healthy control-derived PBMCs were stimulated using sodium iodide, with or without lipopolysaccharide (LPS) for 72 h.

**Results:**

PBMCs from patients with AIT had significantly higher levels of pro-IL-18, pro-IL-1β and NLRP3 than did the PBMCs from the healthy controls (*P* < 0.05). Compared with those from the controls, AIT-derived PBMCs had enhanced levels of active IL-18 and active caspase-1 p20 (*P* < 0.05), whereas their abundance of active IL-1β was similar (*P* > 0.05). In serum, the AIT group had enhanced levels of IL-18 compared with the healthy controls (*P* < 0.05) but had similar levels of IL-1β (*P* > 0.05). NLRP3 expression on CD14^+^ monocytes from AIT patients was significantly augmented compared with the healthy controls (*P*< 0.01). Significantly increased percentages of Th1 and Th17 cells were detected in AIT patients compared with those in the healthy participants (*P* < 0.001). Sodium iodide treatment upregulated NLRP3 expression in PBMCs during 72 h of culture (*P* < 0.001). The percentage of Th1 and Th17 cells in AIT patients increased in an iodine-dependent manner (*P* < 0.01). Iodine had no significant effect on the number of these cells in the healthy control group (*P* > 0.05).

**Conclusion:**

AIT-derived PBMC NLRP3 activity and expression increased. Iodine might regulate the immune and inflammatory response of patients with AIT by activating NLRP3 and promoting Th1 and Th17 cell differentiation.

## Introduction

Autoimmune thyroiditis (AIT), also known as chronic lymphocytic thyroiditis or Hashimoto’s thyroiditis (HT), is an autoimmune thyroid disease (AITD). Its characteristic features include an enlarged thyroid gland, lymphocytic infiltration of the thyroid parenchyma and the presence of thyroid-specific autoantibodies in serum (1). Immune tolerance can break down because of the genetic and environmental factors (2). Helper T1 (Th1) cells are considered the main immune cells of AIT, which can activate cytotoxic T cells and macrophages, destroy thyroid follicular epithelial cells and cause subsequent thyroiditis and thyroid gland damage (3, 4). In recent years, it has been found that helper T17 (Th17) cells can produce interleukin (IL)-17, which significantly enhances the abundance of inflammation-related cytokines, including tumor necrosis factor alpha (TNF-α) and IL-1β. The downstream cytokines have vital functions in AIT development (5). Inflammasomes, which contain certain members of the pattern recognition receptor family (NLR family pyrin domain-containing 1 (NLRP1) and NLRP3), pro-caspase-1, apoptosis-associated speck-like protein or absent in melanoma 2 (AIM2), are intracellular multiprotein complexes that promote inflammation by activating IL-1β and IL-18. These two interleukins regulate CD4 + T cell differentiation into Th1 and Th17 cells, mediate the chemotaxis and proliferation of immune cells and further participate in various autoimmune disease pathogeneses (6, 7, 8). NLRP3, one of the best studied inflammasome components (9), can be activated by environmental stimuli, endogenous risk signals, crystalloids, pathogenic microorganisms and other stimulating factors (10) to participate in the occurrence and development of other inflammatory and autoimmune diseases (4, 6, 11, 12, 13, 14). For example, NLRP3 mediates Th1 and Th17 cell responses in peripheral blood mononuclear cells (PBMCs), a vital step in experimental autoimmune encephalomyelitis development (15). NLRP3 also regulates the differentiation of Th17 cells in rheumatoid arthritis (RA) (16). Increased inflammasome-associated gene expression in PBMCs might promote the induction of Th17 cells in patients with multiple sclerosis (MS) (17). These studies suggested that activated inflammasomes in PBMCs can regulate Th1 and Th17 cell differentiation.

AIT is affected by several environmental factors, including iodine. The incidence rate of AIT in areas with more than adequate/excessive iodine intake is significantly higher than that in areas with adequate iodine intake (18). The classical AIT animal model, NOD.H-2h4 mice, also confirms that iodine is a necessary condition for inducing and aggravating AIT (19). There are three mechanisms that lead to the imbalance of thyroid immune homeostasis (20): excessive iodine overiodizes thyroglobulin and enhances its immunogenicity, iodine directly stimulates immune cell activation and cytokine secretion, and excessive iodine causes oxidative stress injury and cytotoxicity to the thyroid. Previous studies showed that iodine can affect the activation, proliferation and apoptosis of PBMCs in patients with AITD (21). There are many antioxidative mechanisms in normal cells that allow them to avoid oxidative stress damage. When the production of reactive oxygen species (ROS) induced by excessive iodine exceeds the cell scavenging efficiency, the imbalance of cell oxidation/antioxidation leads to DNA damage or cell degradation. The release by cells of damage-associated molecular patterns (DAMPs), such as cytoplasmic DNA fragments, heat shock protein 90 (HSP90) and high mobility protein box 1 (HMGB1) (10), might activate inflammasomes and release cytokines, resulting in changes in the percentage of T cell subsets (6).

In summary, we believe that DAMPs caused by iodine-induced oxidative stress might be the factors that activate inflammasomes in PBMCs, which has not been reported before. Previous studies have demonstrated that patients with AIT have dysregulated serum IL-1β and IL-18 levels (8, 22). Whether abnormal numbers or activation of inflammasomes affects the AIT pathogenesis of PBMCs is unclear. Therefore, herein, we aimed to assess the expression of classical inflammasome markers (NLRP1, AIM2 and NLRP3) in AIT patient-derived PBMCs, their subsequent activation of cytokines and the effect of Th cell differentiation. Furthermore, the effects of iodine on inflammasomes and the activation of inflammasomes on Th cell differentiation were explored in PBMC primary culture.

## Materials and methods

### Participants and their samples

We recruited 50 patients with AIT presenting to the endocrine clinic and 50 healthy controls matched for age and sex at the medical examination center of the First Hospital of China Medical University. The AIT group comprised 50 patients who were positive for serum thyroglobulin antibody (TgAb) or thyroid peroxidase antibody (TPOAb) (TgAb > 115 IU/mL and/or TPOAb > 34 IU/mL) according to the standard values provided by Roche Diagnostics Ltd (Switzerland). The healthy controls (CON) tested negative for TgAb/TPOAb and had no abnormalities on thyroid palpation or B-ultrasound. Participants in both groups were euthyroid. Participants were excluded according to the following criteria: the existence of chronic inflammatory diseases or other autoimmune disorders, e.g., gout, diabetes mellitus, vitiligo, psoriasis, inflammatory bowel disease, Sjogren’s disease, rheumatic disease and systemic lupus erythematosus (SLE); the presence of chronic or acute infections, e.g., HIV infection or viral hepatitis; and patients who were pregnant, had a malignancy or were taking immunomodulatory drugs. The Medical Ethics Committee of the First Hospital of China Medical University provided approval of the study protocols (code: 201520151352). All study participants consented to the procedures in writing.

### Biochemical assessments

Electrochemiluminescent immunoassays were used to assay serum TgAb, free T3 (FT3), TPOAb, thyrotropin (TSH) and free T4 (FT4) levels on a Cobas Elecsys 601 machine using manufacturer-supplied reference ranges (Roche Diagnostics Ltd).

### Culture of PBMCs

PBMCs were isolated using the density gradient method and resuspended in the Roswell Park Memorial Institute (RPMI)1640 complete culture medium (Solarbio Life Science, China) containing 10% fetal bovine serum. The PBMCs were seeded at 2 × 10^6^ cells/well in 6-well culture plates, incubated with lipopolysaccharide (LPS) at 2 μg/mL for 2 h, and then added with different concentrations of sodium iodide (NaI): 0, 5 × 10^−5^, 2 × 10^−4^, 1 × 10^−3^ and 1 × 10^−2^ mmol/L. The PBMCs continued in culture in complete medium for 72 h, with medium-only or LPS wells as the controls.

### Total RNA extraction and quantitative real-time reverse transcription PCR (qRT-PCR)

The TRIzol reagent (#9109, Takara, Japan) was used to extract total RNA from PBMCs. A NanoDrop 2000c spectrophotometer (NanoDrop Technologies, USA) was employed to determine the RNA concentrations. RNA samples with OD_260_/OD_280_ ratios between 1.8 and 2.0 were considered suitable for further experimentation. A 96-well Veriti™ Thermal Cycler (AB Applied Biosystems, Singapore) combined with a 5× PrimeScript RT reagent kit (Takara; #036A) was used to reverse-transcribe 1000 ng of RNA into cDNA. The cDNA was then subjected to quantitative real-time PCR (qPCR) amplification using a LightCycler 480 Real-Time PCR System (Roche) with a SYBR Premix Ex Taq™ II kit (Takara; #820A). Takara also designed and synthesized the primers (see Table 1). We amplified *GAPDH* (encoding glyceraldehyde-3-phosphate dehydrogenase) as the reference control. Samples were tested in duplicate. During amplification, we generated a melting curve to rule out the presence of incorrectly paired products or primer dimers. The relative expression of the target gene’s mRNA was normalized to that of *GAPDH* according to the 2^−*ΔΔC*_T_^ method (23), following correction according to *GAPDH* expression.

**Table 1:** Primer sequences for real-time PCR.

Gene	Sequences (5′ to 3′)
NLRP1	F: CCA​CAA​CCC​TCT​GTC​TAC​ATT​AC; R: GCC​CCA​TCT​AAC​CCA​TGC​TTC
NLRP3	F: GAT​CTT​CGC​TGC​GAT​CAA​CA; R: GGG​ATT​CGA​AAC​ACG​TGC​ATT​A
AIM2	F: CTG​CAG​TGA​TGA​AGA​CCA​TTC​GTA; R: GGT​GCA​GCA​CGT​TGC​TTT​G
CASP1	F: GCC​TGT​TCC​TGT​GAT​GTG​GAG; R: TGC​CCA​CAG​ACA​TTC​ATA​CAG​TTT​C
IL-1β	F: CCA​GGG​ACA​GGA​TAT​GGA​GCA; R: TTC​AAC​ACG​CAG​GAC​AGG​TAC​AG
IL-18	F: CTG​CCA​CCT​GCT​GCA​GTC​TA; R: TCT​ACT​GGT​TCA​GCA​GCC​ATC​TTT​A
GAPDH	F: GCA​CCG​TCA​AGG​CTG​AGA​AC; R: TGG​TGA​AGA​CGC​CAG​TGG​A

### Extraction of total proteins and western blotting

A Minute™ total protein extraction kit for animal cultured cells and tissues (Invent Biotechnologies Inc., USA; #SD-001/SN-002) was used to extract total proteins from PBMCs. A BCA Protein Assay Kit (Beyotime, China; #P0012S) was utilized to estimate the protein concentrations of the samples, which were balanced to 4 μg/μL. The same amounts of proteins in sample buffer were heated for 6 min at 100°C. Aliquots (15 μL) of the samples were subjected to 10% sodium dodecyl sulfate–acrylamide gel electrophoresis. The separated proteins were electrotransferred onto nylon membranes, which were then subjected to blocking in 5% skim milk. The membranes containing the proteins were then maintained at 4°C overnight with rabbit antibodies recognizing human AIM2 (#12948, 1:1000 dilution; Cell Signal Technology, USA), cleaved IL-18 (#sc-7954, 1:200 dilution; Santa Cruz Biotechnology, USA), cleaved caspase-1 (#4199, 1:1000 dilution; Cell Signal Technology), pro-caspase-1 (#2225, 1:1000 dilution; Cell Signal Technology), NLRP1 (#12256-1-AP, 1:1000 dilution; Proteintech Group Inc., USA), pro-IL-1β (#ab156791, 1:2000 dilution, Abcam, UK), cleaved IL-1β (#83186, 1:1000 dilution; Cell Signal Technology), pro-IL-18 (#10663-1-AP, 1:1000 dilution; Proteintech Group Inc.) and NLRP3 (#15101, 1:1000 dilution; Cell Signal Technology). Rabbit antibodies recognizing the internal reference *GAPDH* (sc-25778, 1:1000 dilution; Santa Cruz Biotechnology) were also utilized. Tris-buffered saline–0.1% Tween 20 was used to rinse the membrane three times for 5 min each time before incubation at room temperature for 1 h in goat anti-rabbit IgG linked to horseradish peroxidase (#7074, 1:10,000 dilution; Cell Signaling Technology). After washing, the immunoreactive proteins were detected using enhanced chemiluminescence (#P0018; Beyotime) and imaged by exposure to film. The grayscale values of the protein bands were quantified using the ImageJ 2.0 software (NIH, USA), which also calculated the target protein:GAPDH band intensity ratio.

### Enzyme-linked immunosorbent assay (ELISA)

Commercial ELISA kits, utilized following the supplier’s protocols (#HSLB00D, Human Platinum ELISA for IL-1β, R&D, USA; #BMS267-2, Human IL-18 Platinum ELISA, eBioscience, USA), were employed to assess IL-1β and IL-18 serum levels in duplicate. The absorbance values were plotted against the standard concentrations from the kits to generate a standard curve. On the same plate as the samples, we analyzed positive and blank controls.

### Flow cytometry (FC) analysis

#### Extracellular staining

After culturing for 72 h, the PBMCs were washed, centrifuged, gently mixed and then added into the 100-μL flow tube. Each flow tube received FC block (2.5 μg), placed at room temperature for 10 min, and the cells were stained using surface antibodies: fluorescein isothiocyanate (FITC)-labeled anti-human CD4 monoclonal antibodies (BD Biosciences, USA), phycoerythrin (PE)-labeled anti-human CD14^+^ monoclonal antibodies or their isotype control antibodies (BD Biosciences) and allophycocyanin (APC)-labeled anti-human NLRP3 monoclonal antibodies or their isotype control antibodies (Miltenyi Biotec, Germany). The tubes were incubated in the absence of light for 30 min at 4°C, rinsed once using phosphate-buffered saline (PBS) and tested after resuspension in PBS or follow-up stained with intracellular antibodies.

#### Intracellular staining

A Fix & Perm Cell Permeabilization Kit (BD Biosciences) was used to fix and permeabilize the cells. PBMCs were incubated at room temperature in the absence of light, washed, centrifuged and resuspended. Then, the corresponding intracellular antibodies were added: PE-labeled anti-human IL-17A monoclonal antibodies (BD Biosciences), APC-labeled anti-human interferon gamma (IFN-γ) monoclonal antibodies (BD Biosciences) or isotype control antibodies (BD, USA). PBMCs were incubated at 4°C in darkness for 30 min, washed twice and resuspended in PBS for immediate detection. Data acquisition was performed employing a BD FACSCalibur flow cytometer, and the FlowJo 7.6 software (Tree Star Inc., USA) was utilized for data analysis.

### Statistical considerations

The SPSS 21.0 software (IBM Corp., USA) was utilized to perform statistical analyses. For normally distributed continuous variables, data are presented as the mean ± standard deviation (*x* ± *s*) and were compared employing an independent-sample *t*-test. Comparisons among multiple groups employed one-way analysis of variance (one-way ANOVA), and pairwise comparisons between groups used a least significant distance *t* test. The non-normally distributed or uneven variance data were expressed as the median (*M*) (*P25* and *P75*) and compared utilizing the nonparametric Mann–Whitney *U* test. The correlation between skewed distribution data and other variables was assessed according to the Spearman correlation. Percentages and counts were used to express the categorical variables. Statistical analyses also used the chi-squared test and Fisher’s exact test. Statistical significance was considered at *P* < 0.05.

## Results

### Clinical characteristics of patients and healthy control subjects

Table 2 shows the clinical and demographic clinical characteristics of all participants. Between the groups, cigarette consumption, body mass index (BMI), sex ratio and age did not show significant differences, indicating their similar baselines. Although the participants in the two groups were euthyroid, the TSH levels in serum from patients with AIT were enhanced in comparison with those of the controls (*P* = 0.011). TPOAb and TgAb levels were markedly enhanced in the AIT group compared with those in the healthy control group (*P* < 0.01).

**Table 2:** Demographic and clinical characteristics of AIT patients and the healthy control group.

	HC	AIT	*P* value
*n*	50	50	
Age (year)^a^	41.9 ± 11.4	43.6 ± 12.3	0.461
Sex (F/M)^c^	6/44	3/47	0.295
Smoking^c^	3/47	2/48	0.642
BMI (kg/m^2^)^a^	22.39 ± 3.67	23.13 ± 4.14	0.568
FT3 (pmol/L)^b^	4.23 (4.08–4.66)	4.46 (4.01–4.76)	0.950
FT4 (pmol/L)^a^	14.89 ± 2.14	15.39 ± 2.73	0.314
TSH (mIU/L)^b^	1.69 (1.16–2.38)	2.84 (1.88–4.13)	0.011*
TPOAb (IU/mL)^a^	11.96 ± 10.02	272.79 ± 218.3	0.001^†^
TgAb (IU/mL)^b^	5.74 (1.18–16.7)	211.73 (71.87–407.55)	0.001^†^

^a^ Variables are shown as mean ± S.D. Comparisons among groups were conducted by Student’s *t*-test.

^b^ Variables are shown as median with interquartile range. Comparisons among groups were conducted by Mann–Whitney *U* test.

^c^ Categorical variables are shown as frequencies, and chi-square test was applied for comparisons between groups.

*P<0.05; ^†^*P*<0.01: AIT compared with HC. The normal reference ranges were obtained from the manufacturer (Roche Standard): FT3, 3.10–6.80 pmol/L; FT4, 12.00–22.00 pmol/L; TSH, 0.27–4.20 mIU/L; TPOAb, 0–34 IU/mL; TgAb: 0–115 IU/mL. F, female; M, male; BMI, body mass index; AIT, autoimmune thyroiditis; HC, healthy control.

### Inflammasome component levels in AIT and control PBMCs

**Figure 1:**
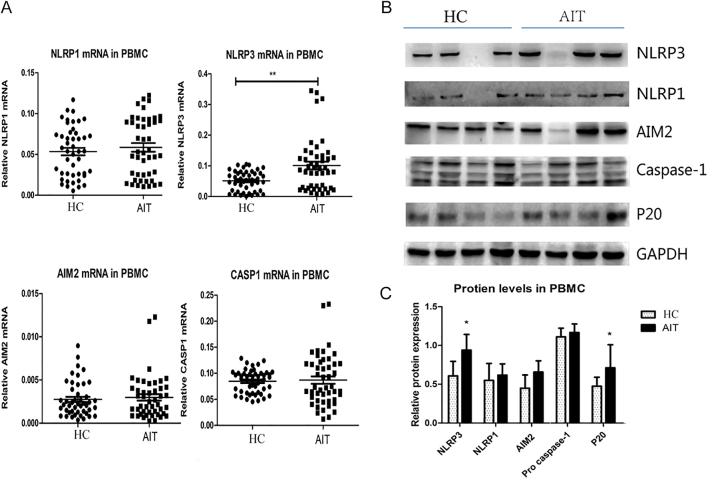
PBMCs from patients with AIT had increased inflammasome component levels. (A) NLRP1, NLRP3, AIM2 and CASP1 mRNA levels in AIT patients and control thyroid tissues (*n* = 50 per group). Representative immunoreactive protein bands (B) and related quantitative determination (C) of the protein levels of NLRP1, NLRP3, AIM2, caspase-1 and caspase-1 p20 in PBMCs from the AIT and HC groups (*n* = 20 per group). GAPDH expression was used for normalization. The columns represent the mean ± SD values. The patient and control data were compared using Student’s *t*-test or Mann–Whitney *U* test. *P < 0.05; **P < 0.01. AIT, autoimmune thyroiditis; HC, healthy control; PMBC, peripheral blood mononuclear cell.

In AIT PBMCs, the mRNA expression of *NLRP3* was markedly augmented compared with that in the healthy control PBMCs (Fig. 1A; *P* < 0.01), whereas *NLRP1*, *AIM2* and *CASP1* showed no differences between the two groups (*P* > 0.05). Consistent with the mRNA expression, we determined increased NLRP3 protein levels in PBMCs from patients with AIT, whereas NLRP1, AIM2 and caspase-1 levels in the AIT group and control group were similar. In the AIT group, the levels of P20, an indicator of inflammasome activity, were higher than those in the healthy controls (*P* < 0.05) (Fig. 1B and C).

### IL-18 and IL-1β levels in patients with AIT patients and healthy controls

**Figure 2:**
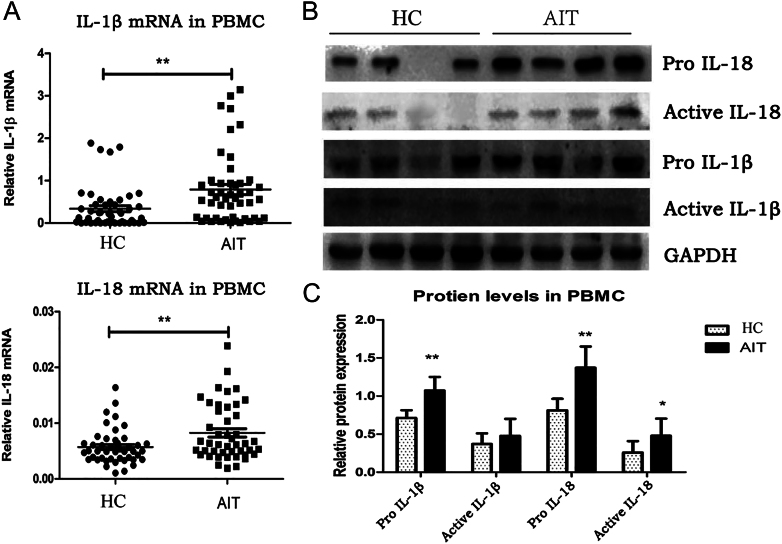
Increased levels and maturation of IL-1β and IL-18 PBMCs from patients with AIT. (A) IL-1β and IL-18 mRNA levels in PBMCs from patients with AIT and HCs (*n* = 50 per group). Representative immunoreactive protein bands (B) and related quantitative determination (C) of pro-IL-18, active IL-18, pro-IL-1β and active IL-1β levels in patient and control PBMCs (*n* = 20 per group). GAPDH expression was used for normalization. The columns represent the mean ± SD values. Patient and control data were compared using Student’s *t*-test or Mann–Whitney *U* test. *P < 0.05; **P < 0.01. AIT, autoimmune thyroiditis; HC, healthy control; PMBC, peripheral blood mononuclear cell.

The pro-IL-1β and pro-IL-18 mRNA and protein levels were significantly higher in patient PBMCs than in the control PBMCs (mRNA = *P* < 0.01; protein = *P* < 0.01; Fig. 2). Active IL-18 (*P* < 0.01) was increased in the PBMCs from patients with AIT relative to that in the controls, whereas active IL-1β levels in the patients and controls were similar (*P* > 0.05).

### IL-18 and IL-1β levels in serum of the AIT group and control group

**Figure 3:**
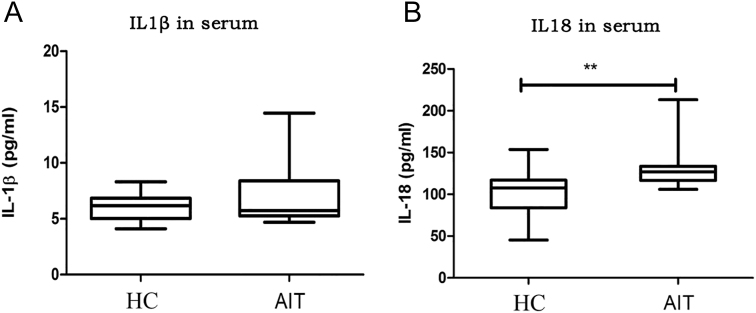
IL-18 and IL-1β levels in PBMCs from patients with AIT. (A) Serum levels of IL-1β in PBMCs from patients with AIT and controls. (B) Serum levels of IL-18 in patient and control PBMCs. The columns represent the mean ± SD values. Mann–Whitney *U*-tests and Student’s *t*-tests were employed to compare the data between the AIT and HC groups. *P < 0.05; **P < 0.01. AIT, autoimmune thyroiditis; HC, healthy control; PMBC, peripheral blood mononuclear cell.

Serum IL-1β and IL-18 levels in the two groups were detected using ELISA (Fig. 3). Compared with that in the controls, IL-18 levels were markedly augmented in the AIT group (*P* < 0.05), whereas IL-1β levels were not (*P* > 0.05).

### Correlation between *NLRP3*, *IL-1β* and *IL-18* mRNA expression in PBMCs and serum autoantibodies

**Figure 4:**
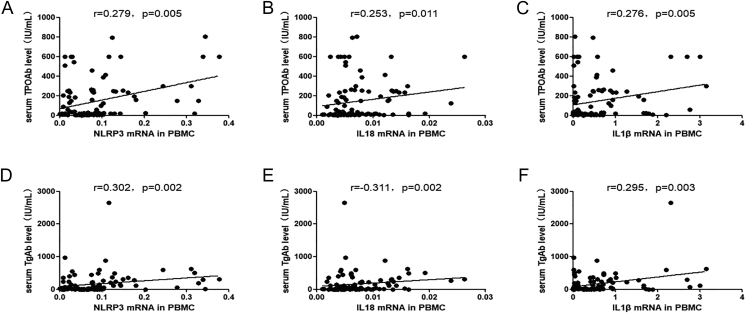
Correlation analysis of inflammasome component mRNA levels in PBMCs and serum autoantibody levels in patients with autoimmune thyroiditis (AIT). Correlation analysis of *NLRP3*, *IL-18* and *IL-1β* mRNA levels in PBMCs and the serum levels of thyroid peroxidase antibody (TPOAb)/thyroglobulin antibody (TgAb) levels (A, B, C, D, E, F). The Spearman rank test was used to carry out bivariate correlation analysis (*r* = the Spearman correlation coefficient). PMBC, peripheral blood mononuclear cell.

*NLRP3*, *IL-1*β and *IL-18* mRNA expressions correlated positively with serum TPOAb and TgAb levels (Fig. 4).

### Increased expression of NLRP3 in monocytes of patients with AIT

**Figure 5:**
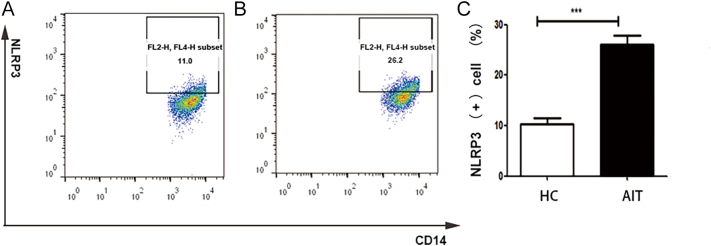
NLRP3 expression on monocytes of HC and AIT groups. (A) HC group, (B) AIT group and (C) the corresponding quantitative analysis (*n* = 30 per group). The columns represent the mean ± SD values. Mann–Whitney *U*-tests and Student’s *t*-tests compared the data between the patients and HCs. *P < 0.05; **P < 0.01; ***P < 0.001. AIT, autoimmune thyroiditis; HC, healthy control.

NLRP3 expression on CD14^+^ monocytes was detected using flow cytometry (Fig. 5). In the AIT group, the expression level of NLRP3 on CD14^+^ monocytes was markedly augmented compared with their levels in the healthy controls (*P* < 0.001).

### Iodine affects NLRP3 mRNA and protein levels and its components on PBMCs

**Figure 6:**
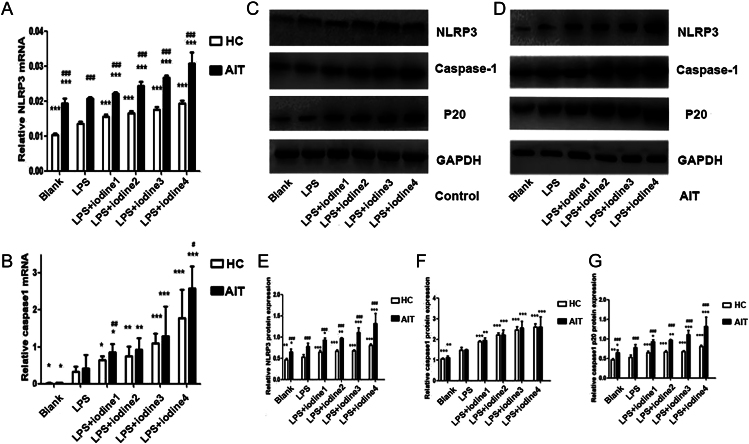
Iodine’s effects on the relative mRNA and protein levels of NLRP3 and its components in PBMCs from the AIT group and the HC group. (A) *NLRP3* mRNA relative expression; (B) *CASP1* mRNA relative expression; (C), (D) representative immunoreactive protein bands in the HC and AIT groups; (E) the relative protein level of NLRP3; (F) caspase-1 relative protein level; (G) the relative protein level of caspase-1 P20 (*P < 0.05; **P < 0.01; ***P < 0.001: in comparison with the lipopolysaccharide (LPS) group. ^#^*P* < 0.05; ^##^*P* < 0.01; ^###^*P* < 0.001: AIT group vs HC group). Different concentrations of iodine: iodide 1: 5 × 10^−5^ mmol/L, iodide 2: 2 × 10^−4^ mmol/L, iodide 3: 1 × 10^−3^ mmol/L and iodide 4: 1 × 10^−2^ mmol/L. AIT, autoimmune thyroiditis; HC, healthy control.

The PBMCs of healthy control and AIT groups were separated and incubated with LPS (24, 25), which can activate CD14^+^ monocytes and trigger the activation of inflammasomes, followed by addition of different concentrations of sodium iodide to the 72-h culture. In the blank group, the LPS group and the group with different iodine concentrations, on the basis of incubation with LPS, *NLRP3* mRNA levels in the patient group were consistently higher compared with those in the control group (*P* < 0.05; Fig. 6).

The results showed that *NLRP3* and *CASP1* mRNA levels in PBMCs of both the healthy control and AIT groups were increased by adding 2 μg/mL LPS alone (*P* < 0.05). On this basis, *NLRP3* and *CASP1* mRNA levels increased according to the iodine concentration (NaI).

In the blank group, the LPS group and the group with different iodine concentrations, on the basis of incubation with LPS, the AIT group protein levels of NLRP3 and caspase-1 p20 were consistently higher compared with those in the healthy controls (*P* < 0.05; Fig. 6).

The results indicated that the *NLRP3*, *CASP1* and *P20* mRNA levels in the PBMCs of both the healthy control group and the AIT group were increased by adding 2 μg/mL LPS alone (*P* < 0.05). On this basis, the *NLRP3*, *CASP1* and *P20* mRNA levels increased in an iodine concentration-dependent manner with the addition of different concentrations of NaI.

### Iodine affects the expression of NLRP3 in monocytes and the percentage of differentiated Th1 and Th17 cells in AIT patients

**Figure 7:**
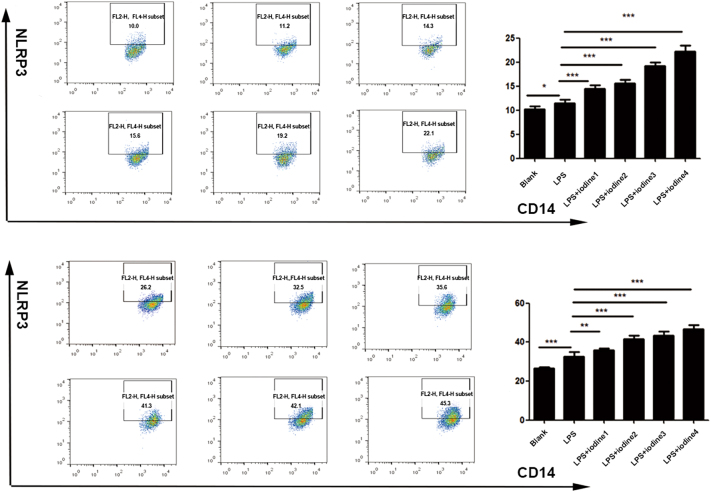
NLRP3 levels in monocytes of the healthy control (HC) group and the autoimmune thyroiditis (AIT) group after lipopolysaccharide (LPS) and sodium iodide stimulation, as determined using flow cytometry. HC group: (A) only culture medium without a stimulant; (B) 2 μg/mL LPS; (C, D, E, F) different concentrations of iodine were added; (G) statistical chart of NLRP3 expression in the monocytes of the six groups (*P < 0.05; **P < 0.01; ***P < 0.001). AIT group: (H) only culture medium without a stimulant; (I) 2 μg/mL LPS; (J, K, L, M) different concentrations of iodine were added; (N) statistical chart of NLRP3 expression in monocytes of the six groups (*P < 0.05; **P < 0.01; ***P < 0.001). Different concentrations of iodine: iodide 1: 5 × 10^−5^ mmol/L, iodide 2: 2 × 10^−4^ mmol/L, iodide 3: 1 × 10^−3^ mmol/L and iodide 4: 1 × 10^−2^ mmol/L.

**Figure 8:**
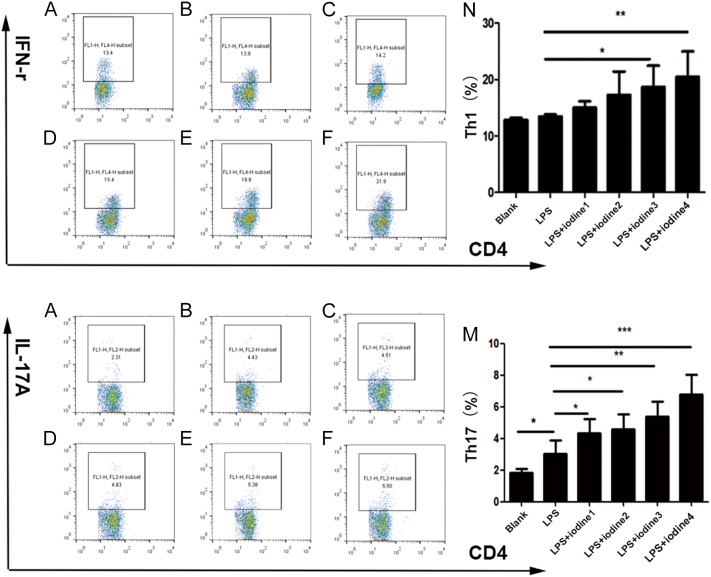
Flow cytometry analysis of the changes in Th1 and Th17 cell differentiation percentage in autoimmune thyroiditis (AIT) monocytes after lipopolysaccharide (LPS) and sodium iodide stimulation. Th1 cell: (A) only culture medium without a stimulant; (B) 2 μg/mL LPS; (C, D, E, F) different concentrations of iodine were added. Th17 cell: (A) only culture medium without a stimulant; (B) 2 μg/mL LPS; (C, D, E, F) different concentrations of iodine were added; (N) statistical chart of Th1 cell differentiation percentage changes in the six groups; (M) statistical chart of the change in the percentage of Th17 cell differentiation in the six groups (*P < 0.05; **P < 0.01; ***P < 0.001). Different concentrations of iodine: iodide 1: 5 × 10^−5^ mmol/L, iodide 2: 2 × 10^−4^ mmol/L, iodide 3: 1 × 10^−3^ mmol/L and iodide 4: 1 × 10^−2^ mmol/L.

In comparison with the blank control, NLRP3 expression and the percentage of Th17 cells in CD14^+^ monocytes were increased after the treatment of PBMCs from patients with AIT with 2 µg/mL LPS (*P* < 0.05) (Table 3, Figs 7 and 8). In addition, the expression level of NLRP3 in CD14^+^ monocytes and the percentage of Th1 and Th17 cells increased in an iodine concentration-dependent manner (Figs 7 and 8). When the PBMCs of the healthy control group were stimulated, the level of NLRP3 in CD14^+^ monocytes could be increased by adding 2 µg/mL LPS alone (*P* < 0.05) (Fig. 7), although the percentage of Th1 and Th17 cells was not significantly different. In the control group, NLRP3 expression on CD14^+^ monocytes increased according to the concentration of iodine (*P* < 0.001; Fig. 7), while there was no significant difference in the percentage of Th1 and Th17 cells (*P* > 0.05).

**Table 3:** Effects of iodine on the expression of NLRP3 in CD14^+^ monocytes and the differentiation percentage of Th1 and Th17 cells of AIT patients stimulated by LPS (*x* ± *s*).

Group	Stimulant	Concentration	NLRP3 (+)	Th1 (%)	Th17 (%)
HC	None		10.25 ± 0.57*	9.03 ± 0.63	1.60 ± 0.34
	LPS	2 μg/mL	11.50 ± 0.76	10.57 ± 1.63	2.07 ± 0.23
	Iodine 1	5 × 10^−5^ mmol/L	14.50 ± 0.70^‡^	10.71 ± 1.31	2.05 ± 0.55
	Iodine 2	2 × 10^−4^ mmol/L	15.62 ± 0.73^‡^	10.48 ± 1.62	2.18 ± 0.08
	Iodine 3	1 × 10^−3^ mmol/L	19.23 ± 0.68^‡^	11.40 ± 1.70	2.34 ± 0.36
	Iodine 4	1 × 10^−2^ mmol/L	22.21 ± 1.24^‡^	10.84 ± 1.45	2.17 ± 0.45
AIT	None		26.67 ± 0.60^‡^	12.86 ± 0.36	1.83 ± 0.27*
	LPS	2 μg/mL	32.60 ± 2.37	13.48 ± 0.34	3.04 ± 0.82
	Iodine 1	5 × 10^−5^ mmol/L	35.90 ± 0.91^†^	15.02 ± 1.15	4.32 ± 0.90*
	Iodine 2	2 × 10^−4^ mmol/L	41.5 ± 1.91^‡^	17.28 ± 4.17	4.58 ± 0.95*
	Iodine 3	1 × 10^−3^ mmol/L	43.43 ± 1.97^‡^	18.70 ± 3.76*	5.36 ± 0.99^†^
	Iodine 4	1 × 10^−2^ mmol/L	46.77 ± 1.99^‡^	20.48 ± 4.51^†^	6.77 ± 1.26^‡^

Different concentrations of iodine: iodine 1: 5 × 10^−5^ mmol/L, iodine 2: 2 × 10^−4^ mmol/L, iodine 3: 1 × 10^−3^ mmol/L and iodine 4: 1 × 10^−2^ mmol/L. Student’s *t*-test or Mann–Whitney *U*-test was used for comparison (*P < 0.05;^†^*P* < 0.01; ^‡^*P* < 0.001: compared with the LPS group).

## Discussion

In this study, we examined the expression characteristics of certain inflammasome factors and their related cytokines in PBMCs isolated from AIT patients. *In vitro* data showed that iodine, as an environmental factor, aggravates AIT, which might lead to the activation of inflammasomes and subsequent alterations in the percentage of T cell subsets, which regulates the immune response (18). Our study revealed that iodine-induced PBMC inflammasome activation and the regulation of Th1 and Th17 cell differentiation might form part of the mechanism of AIT development.

Inflammasomes are protein complexes distributed in the cytoplasm of cells, which can not only mediate pathogen infection but also regulate innate and adaptive immune responses (26). Recent research has investigated the functions of inflammasomes in autoimmune and inflammatory diseases. Downregulated NLRP1 and NLRP3 expression in PBMCs from patients with SLE was observed, which is related to disease activity (27). However, in PBMCs from patients with ankylosing spondylitis, Sjogren’s syndrome and RA, NLRP3 was activated to initiate an inflammatory response (5, 28, 29). In MS-derived PBMCs, NLRP3 expression was significantly increased, and its activation made Th cells express increased levels of pro-inflammatory components (17). Previously, we demonstrated an increase in the number of inflammasomes and downstream cytokines in AIT thyroid glands. These findings, and the enhanced posttranslational modifications of IL-1β, caspase-1 and IL-18, indicated that inflammasomes were overactivated in the AIT thyroid tissue (30). However, it is unclear whether the expression and activation of inflammasomes in PBMCs function in AIT pathogenesis. Here, we observed increased mRNA and protein levels of pro-IL-18, pro-IL-1β and NLRP3 in PBMCs isolated from AIT patients in comparison with those in PBMCs from the healthy controls. Increased levels of P20 and active IL-18, regarded as activation indexes of inflammasomes, were also detected in the PBMCs of patients with AIT, suggesting increased inflammasome activity (31). The mRNA levels of IL-1β and IL-18 in PBMCs showed differences between groups, indicating that the first step (priming) affected the transcription of IL-1β and IL-18 in the AIT group. However, differences in protein levels in PBMCs and cytokine levels in serum were only observed for IL-18, not IL-1β. This suggests that during the second step (activation), the AIT group only affected the release of mature IL-18. Consistent with our results, highly abundant and overactivated inflammasomes in PBMCs play a role in other autoimmune diseases (5, 16, 17). However, in different autoimmune diseases, different inflammasomes participate in the pathogenesis of the disease in target tissues and immune cells, which might be related to disease specificity. In addition, in this study, PBMC and serum levels of IL-1β were similar between the two groups. The level of serum IL-18 in the AIT group was elevated compared with that in the healthy controls, which suggested an increase in the biological activity of IL-18. Indeed, active IL-18 could induce Th17- and Th1-mediated adaptive immune responses (32). Moreover, *IL18*, *IL1B* and *NLRP3* mRNA levels correlated with the levels of TPOAb and TgAb in serum, suggesting that NLRP3 in PBMCs might be a potential biomarker of AIT.

PBMCs comprise lymphocytes (T and B lymphocytes), monocytes and plasma cells. It is suspected that the activation of inflammasomes in CD14^+^ monocytes might have a vital function in the adaptive immunity of AIT. Indeed, our experiments verified this suspicion. We found that CD14^+^ inflammatory mononuclear cells were the main cells that highly expressed NLRP3 in AIT, which indicated that NLRP3 is involved in AIT immune regulation through antigen presentation (33). The innate immune response, as a connection point of genetic susceptibility and environmental risk factors, provides a possible direction for the next process of adaptive immune response, leading to autoimmune damage.

It is reported that inflammasomes promote the posttranslational modification of cytokines into their active forms via caspase-1, which mediates the chemotaxis and proliferation of immune cells, regulates the differentiation of CD4^+^ T cells into Th1 and Th17 cells and participates in the mechanism of occurrence and development of a variety of autoimmune diseases (32). In autoimmune diseases, including autoimmune encephalomyelitis, RA and MS, Th17 cell differentiation from PBMCs plays a leading role in autoimmunity and the activated inflammasomes in PBMCs can regulate Th1 and Th17 differentiation (34). However, the immune responses mediated by Th1 and Th17 cells can act on subsequent thyroiditis and thyroid gland damage. It has been proposed that Th17 cells play a particularly important role in the development of AIT (35). For example, herein, the percentage of pro-inflammatory Th1 and Th17 cells in patients with AIT was significantly higher than in the healthy controls, which agreed with the results of a previous study (36). Further stimulation by LPS of AIT-derived PBMCs *in vitro* led to upregulation of NLRP3 expression in CD14^+^ monocytes and the differentiation of Th cells into inflammatory Th1 and Th17. This suggested that active NLRP3 in patients with AIT might partially regulate Th1 and Th17 differentiation to mediate AIT autoimmunity. Interestingly, PBMCs and CD14^+^ monocytes from patients with AIT treated with LPS had higher expression of NLRP3 than the PBMCs from the healthy controls and promoted the upregulation of Th1 and Th17 cells differentiation. However, after PBMCs and CD14^+^ monocytes from healthy controls were stimulated, the expression of NLRP3 increased only slightly, and the percentage of Th1 and Th17 cells showed no significant change. This suggested that NLRP3 expression in AIT patient-derived PBMCs resulted in a higher potential for their differentiation into Th1 and Th17 cells. Although the expression of NLRP3 in PBMCs from the healthy control group was upregulated after LPS induction, it did not cause a shift in the balance of Th cell differentiation. On the one hand, it might be the effect of the stimulation concentration; on the other hand, it might indicate that PBMCs without a disease background only change the expression level of NLRP3, which is not sufficient to alter the immune cell differentiation balance to regulate autoimmunity.

Iodine is a trace element in the human body and is one of the important environmental factors of AIT (37, 38). Recently, an observational study has shown that in different populations, excess iodine intake enhances the incidence and prevalence of AIT (39). Research suggested that excess iodine promoted aberrant pyroptosis in thyroid follicular cells (TFCs) by activating NLRP3, possibly pivotally predisposing them to develop HT (40). Research has focused on iodine, thyroid cells and pyroptosis. We are more interested in PBMCs of AIT and the immunological mechanism of iodine in AIT. Excessive iodine can promote Th17 cell infiltration (41), and PBMCs from patients with AIT had abnormal proliferation and activation under the action of iodine (21). However, the specific immunological mechanism of thyroid autoimmunity caused by iodine excess is unclear; therefore, we speculated that excessive iodine stimulates immune cells (PBMCs), and DAMPs produced by oxidative stress might activate inflammasomes to initiate an immune response (42, 43). Other studies have shown that, on the one hand, LPS can activate CD14^+^ monocytes, release pro-inflammatory cytokines and chemokines and participate in the inflammatory response, leading to tissue and organ damage (24). On the other hand, LPS, as the main exogenous stimulant, can induce ROS production and trigger the activation of NLRP3 and caspase-1 (25, 44, 45, 46). However, under conditions of LPS triggering, whether iodine, as an environmental risk factor, can activate inflammasomes has not been reported. Therefore, a cross-sectional case–control study was used to collect PBMCs from patients with AIT and the healthy control group. The PBMCs were stimulated *in vitro* with 2 µg/mL LPS and a gradient concentration NaI for 72 h. Our findings demonstrated that NLRP3 expression in PBMCs and CD14^+^ monocytes was upregulated in an iodine concentration-dependent manner, regardless of the disease background. This suggested that iodine can induce the upregulation of inflammasomes on PBMCs and CD14^+^ monocytes to different degrees. However, this effect is more obvious in the background of AIT disease. In addition, the induced changes in the Th1 and Th17 cell differentiation percentage in PBMCs with disease background were iodine concentration dependent, but this was not observed in PBMCs from the healthy controls. The possible reason is that healthy PBMCs only change the number of inflammasomes and their components under the effect of iodine. However, this is not sufficient to cause the differentiation of immune cells to regulate autoimmunity. As an environmental factor, iodine could aggravate AIT and participate in the process of autoimmune diseases by activating inflammasomes. Whether other immune pathways are involved needs further study.

This study has some limitations. First, this was an *in vitro* study and, thus, it is necessary to further explore whether the same results can be reproduced in *in vivo* animal experiments. Second, the cross-sectional study results did not allow us to explain the different dynamic pathogenicity of inflammasomes in AIT at different stages. In the future, more specific conclusions might be achieved by observing the development of the disease in animal models at continuous time points. In addition, iodine, as an environmental factor of AIT, might promote Th cell differentiation by activating NLRP3. Whether other immune pathways are involved requires further study. Further study of other inflammasomes, various cytokines and activating factors might help explain the interaction between them.

To conclude, our findings suggested that the autoimmune response centered on inflammasomes mediates the development of AIT. Inflammasomes might be used as biomarkers of AIT to monitor the disease. Inflammasomes, as innate immunosensors, regulate the differentiation of Th1 and Th17 cells through caspase-1, which mediates posttranslational modification and IL-18 and IL-1β activation, which influences the over-activated inflammation. Iodine, an environmental risk factor, might participate in the inflammatory and autoimmunity process of AIT by activating inflammasomes.

## Declaration of interest

The authors declare that there is no conflict of interest that could be perceived as prejudicing the impartiality of the work.

## Funding

This work was supported by the National Natural Science Foundation of Chinahttps://doi.org/10.13039/501100001809 (grant nos 82270836, U1508219 and 82300885).

## Author contribution statement

ZS, WT, YW and QG conceived and designed the research. YW, QG and YLiu were responsible for subject recruitment and collection of specimens. YW, QG and CF performed the experiments. ZS, WT, XG, HG, YLi and WS provided reagents, materials, instruments and analysis tools. ZS, YW and QG analyzed the data and wrote the manuscript. All the authors read and approved the final manuscript.

## Data availability

The data that support the findings of this study are available from the corresponding author upon reasonable request.

## Ethics approval

All research procedures were approved by the Medical Ethics Committee of the First Hospital of China Medical University, and all participants provided their informed and written consent.
